# Green manuring combined with rice straw return enhances secondary and micronutrients in rice grains in Southern China

**DOI:** 10.3389/fpls.2026.1826037

**Published:** 2026-04-15

**Authors:** Cuilan Wei, Bingshuai Cao, Shun Li, Jintao Du, Songjuan Gao, Pinzhu Qin

**Affiliations:** 1 College of Environment and Ecology, Jiangsu Open University, Nanjing, China; 2 Nanjing Institute of Environmental Sciences, Ministry of Ecology and Environment, Nanjing, China; 3 College of Environmental Engineering, Suzhou Polytechnic institute of Agriculture, Suzhou, Jiangsu, China; 4 College of Resources and Environmental Sciences, Nanjing Agricultural University, Nanjing, Jiangsu, China

**Keywords:** milk vetch, rice grain, rice straw, secondary nutrients, trace elements

## Abstract

Green manure (GM)-rice rotation is an effective practice for yield-increasing and soil-fertilizing in southern China, while the effects of GM, and the co-utilization with rice straw return on secondary and micronutrients in rice grain remains unclear. This study systematically investigated the regulatory effects of the co-utilization of GM and rice straw combined with reduced nitrogen (N) fertilizer application on grain secondary and micronutrients based on a field experiment in the southern Jiangsu rice-growing area. Results showed that the five-year average rice yield under GM increased by 5.65% relative to that under winter fallow (WF). The co-utilization of GM and rice straw with 40% reduction of N fertilizer (GMS_N60) was identified as the optimal mode. It achieved a rice yield increased by 16.87% compared with the WF with straw return and conventional N fertilization (WFS_N100), and the accumulation of N, phosphorus (P), and potassium (K) improved simultaneously. Green manuring promoted the uptake of secondary and micronutrients in rice grain and silicon (Si) in straw. The average contents of calcium (Ca), iron (Fe), copper (Cu), molybdenum (Mo), and selenium (Se) in rice grain increased by 8.08%, 17.75%, 3.99%, 13.56%, and 20.25%, respectively, and the rice straw Si content increased by 5.24% under GM relative to that under WF. The contents of zinc (Zn) and Mo under GMS_N60 increased by 8.38% and 19.12%, respectively, relative to those under WFS_N100. Correlation analysis indicated that GM facilitated the synergistic absorption of magnesium (Mg), manganese (Mn), and Zn. This study elucidates the roles of GM in stabilizing and increasing rice yields and enhancing the nutritional quality of rice, providing important support for high-yield and high-quality rice production in southern China.

## 1 Introduction

Rice serves as the staple food for more than half of the global population. As the world’s largest rice producer, China considers the synergistic enhancement of yield and quality as a core objective for ensuring food security (Fairhurst and Dobermann, 2002). Historically, intensive rice production systems oriented toward high yields have relied heavily on chemical fertilizers. Although this practice boosts production, it has resulted in continuous soil degradation and a concomitant decline in rice quality (Liu and Li, 2023; Zhu et al., 2017), thereby severely hindering the high-quality development of China’s rice industry. Currently, with sustained economic growth and rising living standards, public demand has shifted from mere food sufficiency (“having enough”) to superior quality (“eating well”). Consequently, achieving high quality while maintaining high and stable yields has emerged as a frontier topic at the intersection of crop science, soil science, and nutritional health (Wan, 2021).

The secondary and micronutrients in rice grains are essential plant nutrients, and exert considerable influence on various physiological processes, including photosynthesis, energy metabolism, and protein synthesis (Li et al., 2022), which are critical determinants of nutritional quality. Elements such as calcium (Ca), magnesium (Mg), copper (Cu), zinc (Zn), iron (Fe), manganese (Mn), molybdenum (Mo), and selenium (Se) not only participate in various physiological and metabolic processes during grain development but also directly influence the nutritional value for human consumption (Montanaro et al., 2015; Li et al., 2022; Becker and Asch, 2005; Yin et al., 2016; Cakmak and Kutman, 2018). Adequate accumulation of these essential elements contributes to balanced mineral nutrition, while deficiencies or imbalances may compromise both grain quality and health benefits. Therefore, understanding the factors that regulate the uptake and distribution of medium and micronutrients in rice is essential for producing nutritionally superior grains.

In agricultural production, various agronomic measures and fertilization methods considerably influence the content of secondary and micronutrients in rice grains by regulating soil physicochemical properties, nutrient availability, and plant transport efficiency. Utilizing winter fallow (WF) fields for green manure (GM) cultivation in the southern rice-growing regions of China is a vital strategy for improving soil fertility and ensuring stable and high rice yields (Gao et al., 2023). GM can introduce substantial amounts of N into agroecosystems through biological N fixation. Studies have indicated that after incorporation into the soil, GM releases various nutrients for absorption and utilization by subsequent crops. For instance, in the arid region of Longzhong, soil-available Mn, Cu, and Zn contents increased considerably by 18.8%, 22.5%, and 14.3%, respectively, after GM incorporation (Song et al., 2023). In plots planted with hairy vetch and ryegrass, soil-exchangeable Ca content increased by more than 13.13% and 23.56%, respectively, whereas exchangeable Mg content increased by more than 13.85% and 20.51%, respectively (Guo et al., 2025). Total soil Fe, Mn, and Zn contents in spring rapeseed plots were 9.31%, 5.71%, and 9.97% higher, respectively, than those in plots under spring fallow treatment (Bei et al., 2023). Straw contains large amounts of N, phosphorus (P), and potassium (K) that can effectively supplement soil nutrients and ameliorate the environment and physicochemical properties of the soil (Hua et al., 2025). Furthermore, straw enhances soil microbial activity by increasing soil organic matter content, thereby further promoting the cycling and reuse of inherent trace elements in soil and consequently improving the absorption and accumulation of these nutrients by crops. Si accumulation in rice straw plays a crucial role in determining grain quality by influencing both physiological resilience. Thus, maintaining appropriate silicon levels in rice plants is essential for producing high-quality grains (Marxen et al., 2016). Former studies found that straw return effectively improved soil Si nutrition supply (Hughes et al., 2020) and enhance Se immobilization (Dhillon et al., 2007). Straw decomposition releases Zn, while the increase in organic matter facilitates the transformation of native soil Zn into available forms (Kalbitz et al., 2003). Additionally, straw return significantly increases exchangeable Mn and Cu levels (Wang, 2016).

Milk vetch is a traditional GM crop in the rice-growing regions of southern China. When incorporated into the soil at the full-bloom stage, its decomposition supplies abundant nutrients for the growth and development of rice (Yang et al., 2021). The combined utilization of milk vetch and rice straw represents a win–win strategy that balances GM production with straw return and is widely applied in southern China. Studies have found that the combined return of milk vetch and rice straw leverages the respective advantages of milk vetch and rice straw well, yielding good outcomes in terms of yield increase and fertilizer reduction (Wei et al., 2025; Zhou et al., 2024). Existing research indicates that the cultivation and utilization of GM considerably enhance the nutritional quality of rice. Specifically, compared with the sole application of chemical fertilizers in WF fields, the combined application of GM and chemical fertilizers (with a 40% N reduction) increased total amino acid and essential amino acid contents in rice by 62.6% and 30.7%, respectively (Gao et al., 2023). However, the effect of GM on trace elements in rice grains remains unclear. On the basis of a long-term field experiment on the combined utilization of milk vetch and rice with reduced N application established in the southern Jiangsu rice-growing region, this study systematically evaluated the individual and interactive effects of winter-planted milk vetch, rice straw return, and N reduction on trace elements in rice. This work aims to provide theoretical support for enhancing the quality and efficiency of rice production and promoting the resource utilization of rice straw in the Southern Jiangsu region.

## 2 Materials and methods

### 2.1 Experimental site

The experimental site was located in Jingqiao Town Lishui District, Nanjing, Jiangsu Province (31.45°N, 119.06°E). It is situated on the Taihu Lake Plain of the Yangtze River Delta and features a subtropical monsoon climate. It has a mean annual temperature of 15.5°C, annual sunshine duration of 2146 h, annual precipitation of 1037 mm, and average frost-free period of 257 days. The soil in the study area is classified as yellowish sandy soil. The soil pH (1:2.5, soil/water) prior to the initiation of the experiment was 5.96. The contents of soil organic carbon and total N in the plow layer were 25.73 and 1.47 g/kg, respectively, whereas those of available P and K were 13.4 and 120.9 mg/kg, respectively.

### 2.2 Experimental design and implementation

The long-term field experiment was initiated in 2019 and consisted of 12 treatments, as follows: (1) winter fallow (WF) + no straw return + no N fertilizer (WF_N0); (2) green manure milk vetch (GM) + no straw return + no N fertilizer(GM_N0); (3) WF + straw return + no N fertilizer (WFS_N0); (4) GM + straw return + no N (GMS_N0); (5) WF + no straw return + 60% conventional N (WF_N60); (6) GM + no straw return + 60% conventional N (GM_N60); (7) WF + straw return + 60% conventional N (WFS_N60); (8) GM + straw return + 60% conventional N (GMS_N60); (9) WF + no straw return + 100% conventional N (WF_N100); (10) GM + no straw return + 100% conventional N (GM_N100); (11) WF + straw return + 100% conventional N (WFS_N100); and (12) GM + straw return + 100% N (GMS_N100). Each treatment was replicated four times. The field plots measured 20 m^2^ (4 m × 5 m) and were arranged in a completely randomized block design.

The milk vetch variety used was ‘Yijiangzi’ and the midseason rice variety employed was ‘Jingliangyou Huazhan’. Rice was transplanted in early to mid-June each year and harvested at the end of September. Milk vetch was sown in early October of each year and incorporated into the soil at the full-bloom stage in mid-April of the following year. The application rate of fresh milk vetch biomass was 15 t/ha. The N fertilizer used was urea (46% N), the P fertilizer was calcium superphosphate (12% P_2_O_5_), and the K fertilizer was potassium chloride (60% K_2_O). The N fertilizer was split between basal and tillering applications at a ratio of 5:5, whereas the P and K fertilizers were applied entirely as basal fertilizer. The N application rates for the N100 and N60 treatments were 200 and 120 kg N/ha, respectively, and the N0 treatment received no N fertilizer. The application rates of P and K fertilizers were the same for all treatments and were 75 kg P_2_O_5_/ha and 100 kg K_2_O/ha, respectively.

### 2.3 Sample collection and determination of indicators

Rice yield was determined at the maturity stage, and each plot was harvested individually. Three rice hills were randomly sampled from each plot. After their roots were separated from the soil, the plants were washed, and their shoots and roots were separated. The samples were de-enzymed at 105°C for 30 min, then dried to a constant weight at 70°C. The dried samples were weighed, ground, and stored for analysis (grains and straw were separated for plant samples at the maturity stage, and yield components were determined).

Plant samples were digested by using the concentrated sulfuric acid–hydrogen peroxide method. According to the characteristics of each element, the contents of N, P, K, Ca, Mg, Cu, Zn, Fe, Mn, Se, and Mo in rice grains, as well as the Si content in rice straw, were determined. Total N, P, and K were determined through the Kjeldahl method, the vanadium–molybdenum yellow colorimetric method, and flame photometry, respectively (Bao, 2000). The contents of Ca, Mg, Cu, Zn, Fe, and Mn in rice grains were determined via microwave digestion followed by inductively coupled plasma mass spectrometry (GB 5009.268-2016) (National Food Safety Standard of the People's Republic of China, 2016). Qualification was based on the specific mass numbers of elements, and quantification was performed by using the external standard method, where the ratio of the mass spectrometry signal intensity of the analyte to that of an internal standard is proportional to the concentration of the analyte. Se in rice grains was determined through atomic fluorescence spectrometry (National Food Safety Standard of the People's Republic of China, 2017). After acid-heat digestion, Se(VI) was reduced into Se(IV) in a 6 mol/L hydrochloric acid medium. By using sodium borohydride as a reductant, Se(IV) was reduced into hydrogen selenide, which was carried by argon gas into an atomizer for atomization. Under irradiation from a Se hollow cathode lamp, ground-state Se atoms were excited to a high-energy state. Upon returning to the ground state (de-excitation), they emitted fluorescence of characteristic wavelengths. Fluorescence intensity was directly proportional to Se content, which was quantified through comparison with a standard series. Si in rice straw was determined by employing the Si–Mo blue colorimetric method (Dai et al., 2005). A 0.1 g plant sample was weighed into a 15 mL high-pressure resistant plastic centrifuge tube and added with 50% sodium hydroxide solution. The tube was capped, vortexed for mixing, then sterilized at 121°C in an autoclave for 30 min. After being naturally cooled and subjected to pressure release, the mixture was diluted to 50 mL with water, and its absorbance was measured at 700 nm.

### 2.4 Data processing

Analysis of variance and correlation analysis were performed by using SAS 8.1 software to evaluate the effects of different treatments on rice yield, nutrient (N, P, and K) accumulation, and soil physicochemical properties. Multiple comparisons were conducted by applying the least significant difference method at a significance level of P < 0.05. Figures were created by using Origin 2018.

## 3 Results and analysis

### 3.1 Rice yield and nutrient content under the co-utilization of milk vetch and rice straw

The average grain and straw yields under different treatments over the past five years are shown in **Table 1**. The average grain and straw yields under GM treatments had increased by 5.65% and 10.63% compared to WF treatments respectively. The average grain yields under GM and WF treatments without straw return increased with increasing N application. Under rice straw return, the highest average grain yield was observed under N60. Grain yield was highest under GMS_N60, reaching 10.1 t/ha, and was 16.87% (*p* < 0.05) and 7.07% higher than those under WFS_N100 and GMS_N100, respectively. Notably, no significant difference in grain yield was found between GMS_N0 and WFS_N100, indicating that stable yield could be achieved without any N fertilizer application under the combined return of milk vetch and rice straw (**Table 1**). Under treatments without straw return, the variation trend of average straw yield was consistent with that of grain yield. The average straw yield over the five years under GM_N60 had increased by 10.53% relative to that under WF_N100. Under rice straw return conditions, the straw yield under GMS_N60 had increased by 8.87% compared to WFS_N100.

**Table 1:** Average grain and straw yields from 2019 to 2024.

Treatments	Grain yield (kg/ha)		Straw yield (kg/ha)	
	Without straw	With straw	Without straw	With straw
WF_N0	8019 ± 423 b	8138 ± 469 c	9094 ± 740 c	10200 ± 412 b
GM_N0	8069 ± 480 b	8269 ± 361 bc	10350 ± 815 bc	10800 ± 400 b
WF_N60	8925 ± 286 ab	8925 ± 162 abc	11400 ± 106 abc	13031 ± 1018 a
GM_N60	9525 ± 469 a	10058 ± 458 a	13650 ± 584 a	13200 ± 1118 a
WF_N100	9342 ± 120 a	8606 ± 145 bc	12350 ± 565 ab	12125 ± 319 ab
GM_N100	9575 ± 510 a	9394 ± 608 ab	14044 ± 1653 a	13406 ± 1129 a

Data are presented as the mean ± standard error of 4 replicates. Different letters within the same column indicate significant differences (*p* < 0.05).

The results for N, P, and K nutrient contents and accumulation in rice grains under different treatments over the past five years are shown in **Table 2**. No significant differences in N content were found among treatments, whereas N accumulation under GM treatments was 2.03% higher than that under WF treatments. Under rice straw return conditions, N accumulation was highest under GMS_N60, increasing by 14.21%, 8.42%, 13.98%, 6.69%, and 7.29% relative to those under WFS_N0, GMS_N0, WFS_N60, WFS_N100, and GMS_N100, respectively. Although P content under GM treatments did not significantly differ from that under WF treatments, P accumulation had increased by 6.15%. The average P content under straw return treatments was 0.85% higher than that under treatments without straw return. Under treatments without straw return, P accumulation increased with increasing N levels, reaching 29.8, 34.3, and 35.4 kg/ha at N0, N60, and N100, respectively. Under straw return conditions, P accumulation was highest under GMS_N60 and had increased by 19.00% and 9.77% relative to those under WFS_N100 and GMS_N100, respectively. K content showed no significant differences among different treatments. K accumulation increased with rising N levels under treatments without straw return. Under straw return, K accumulation was highest under GMS_N60, reaching 38.1 kg/ha, and was 9.17% and 19.44% higher than those under WF_N100 and WFS_N100, respectively.

**Table 2:** Nitrogen (N), phosphorus (P), and potassium (K) contents and accumulations in rice grains in 2024.

Treatments	N content (g/kg)	N uptake (kg/ha)	P content (g/kg)	P uptake (kg/ha)	K content (g/kg)	K uptake (kg/ha)
Without Straw						
WF_N0	13.35 ± 0.55 a	107.1 ± 7.5 ab	3.65 ± 0.07 a	29.3 ± 2.1 c	3.68 ± 0.05 a	29.6 ± 1.8 c
GM_N0	12.91 ± 0.34 a	104.1 ± 6.3 b	3.77 ± 0.06 a	30.4 ± 2.1 bc	3.75 ± 0.11 a	30.3 ± 2.1 bc
WF_N60	11.61 ± 0.34 a	103.6 ± 4.2 b	3.78 ± 0.03 a	33.8 ± 1.1 abc	3.81 ± 0.03 a	34.0 ± 1.2 abc
GM_N60	11.84 ± 0.62 a	112.3 ± 5.1 ab	3.65 ± 0.01 a	34.8 ± 1.6 ab	3.62 ± 0.01 a	34.5 ± 1.6 ab
WF_N100	13.36 ± 0.78 a	124.8 ± 7.2 a	3.71 ± 0.08 a	34.6 ± 0.5 ab	3.74 ± 0.07 a	34.9 ± 0.3 ab
GM_N100	11.87 ± 0.72 a	113.5 ± 8.4 ab	3.78 ± 0.02 a	36.2 ± 2.0 a	3.80 ± 0.03 a	36.4 ± 2.0 a
With Straw						
WF_N0	12.20 ± 0.51 a	99.2 ± 6.7 a	3.88 ± 0.04 a	31.5 ± 1.6 b	3.88 ± 0.07 a	31.5 ± 1.3 bc
GM_N0	12.61 ± 0.34 a	104.5 ± 6.8 a	3.78 ± 0.06 ab	31.2 ± 1.3 b	3.76 ± 0.07 a	31.0 ± 1.3 c
WF_N60	11.10 ± 0.59 a	99.4 ± 7.2 a	3.63 ± 0.06 b	32.4 ± 1.0 b	3.68 ± 0.05 a	32.8 ± 1.0 bc
GM_N60	11.26 ± 0.17 a	113.3 ± 5.7 a	3.80 ± 0.03 ab	38.2 ± 1.8 a	3.79 ± 0.03 a	38.1 ± 1.8 a
WF_N100	12.35 ± 0.52 a	106.2 ± 3.9 a	3.73 ± 0.04 ab	32.1 ± 0.5 b	3.71 ± 0.05 a	31.9 ± 0.4 bc
GM_N100	11.24 ± 0.50 a	105.6 ± 8.6 a	3.71 ± 0.04 ab	34.8 ± 2.1 ab	3.77 ± 0.06 a	35.4 ± 2.3 ab

Data are presented as the mean ± standard error of 4 replicates. Different letters within the same column indicate significant differences (*p* < 0.05).

### 3.2 Secondary nutrients in rice grains under the co-utilization of milk vetch and rice straw

**Figure 1:**
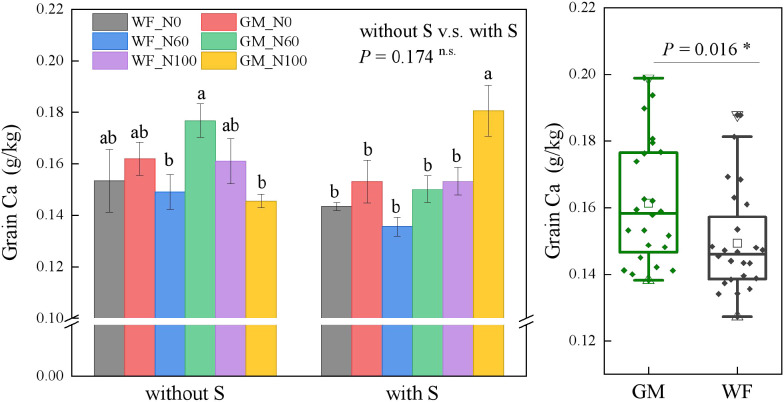
Rice grain calcium (Ca) content under the Co-utilization of Milk Vetch and Rice Straw. Note: Without S and with S represent without rice straw return and with rice straw return, respectively. WF_N0, WF_N60, and WF_N100 represent winter fallow with0%, 60%, and 100% conventional N fertilizer, respectively. GM_N0, GM_N60, and GM_N100 represent green manuring with 0%, 60%, and 100% conventional N fertilizer, respectively (*n* = 4). GM represents the combination of all 6 treatments with green manure, and WF represents the combination of all 6 treatments without green manure (*n* = 24). In the box figures, the solid line and dot within each box represent the median and mean values, respectively. The top and bottom edges represent the 75th and 25th percentiles, respectively; the top and bottom error bars represent the 95th and 5th percentiles, respectively; and the top and bottom triangles represent the 99th and 1st percentiles, respectively. * represents *p < 0.05*, ** represents *p < 0.01.* n.s. represent no significant difference (*p* > 0.05).

The analysis results illustrate that the average Ca content in rice grains under GM treatments had significantly increased by 8.08% (*p* < 0.05) compared with that under WF treatments significantly. Without rice straw returning, the Ca content in rice grains under GM treatments was 4.47% higher than that under WF treatments. The Ca content under GM_N60 had increased by 18.54% and 21.44% relative to those under WF_N60 and GM_N100 (*p* < 0.05), respectively (**Figure 1**). Under straw return, GMS_N60 resulted in an increase of 10.72% compared with WFS_N60, and GMS_N100 resulted in an increase of 17.87% compared with WFS_N100 (*p* < 0.05) (**Figure 1**).

**Figure 2:**
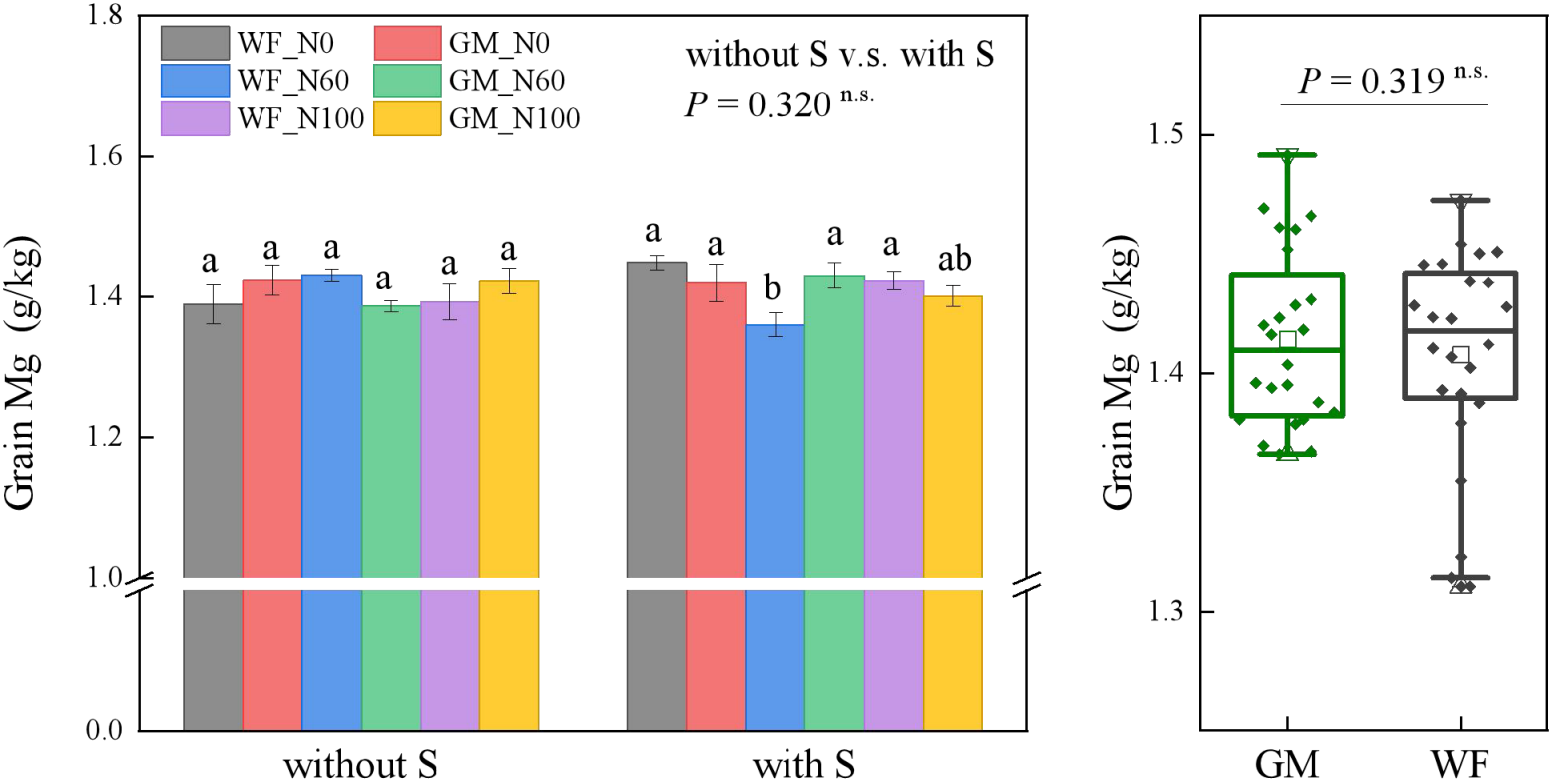
Rice grain magnesium (Mg) content under the Co-utilization of Milk Vetch and Rice Straw. Note: Without S and with S represent without rice straw return and with rice straw return, respectively. WF_N0, WF_N60, and WF_N100 represent winter fallow with0%, 60%, and 100% conventional N fertilizer, respectively. GM_N0, GM_N60, and GM_N100 represent green manuring with 0%, 60%, and 100% conventional N fertilizer, respectively (*n* = 4). GM represents the combination of all 6 treatments with green manure, and WF represents the combination of all 6 treatments without green manure (*n* = 24). In the box figures, the solid line and dot within each box represent the median and mean values, respectively. The top and bottom edges represent the 75th and 25th percentiles, respectively; the top and bottom error bars represent the 95th and 5th percentiles, respectively; and the top and bottom triangles represent the 99th and 1st percentiles, respectively. * represents *p* < 0.05, ** represents *p* < 0.01. n.s. represent no significant difference (*p* > 0.05).

The average Mg content in rice grains did not significantly differ between GM and WF treatments nor between treatments with and without straw return. In the absence of straw return, the Mg content under GM_N0 had increased by 2.47% compared with that under WF_N0. Moreover, Mg content under GM_N100 had increased by 2.14% compared with that under WF_N100. Under straw return, the Mg content in rice grains under GMS_N60 had increased by 5.19% and 2.10% compared with those under WFS_N60 and WFS_N100, respectively (**Figure 2**).

### 3.3 Trace element in rice grains under the co-utilization of milk vetch and rice straw

**Figure 3:**
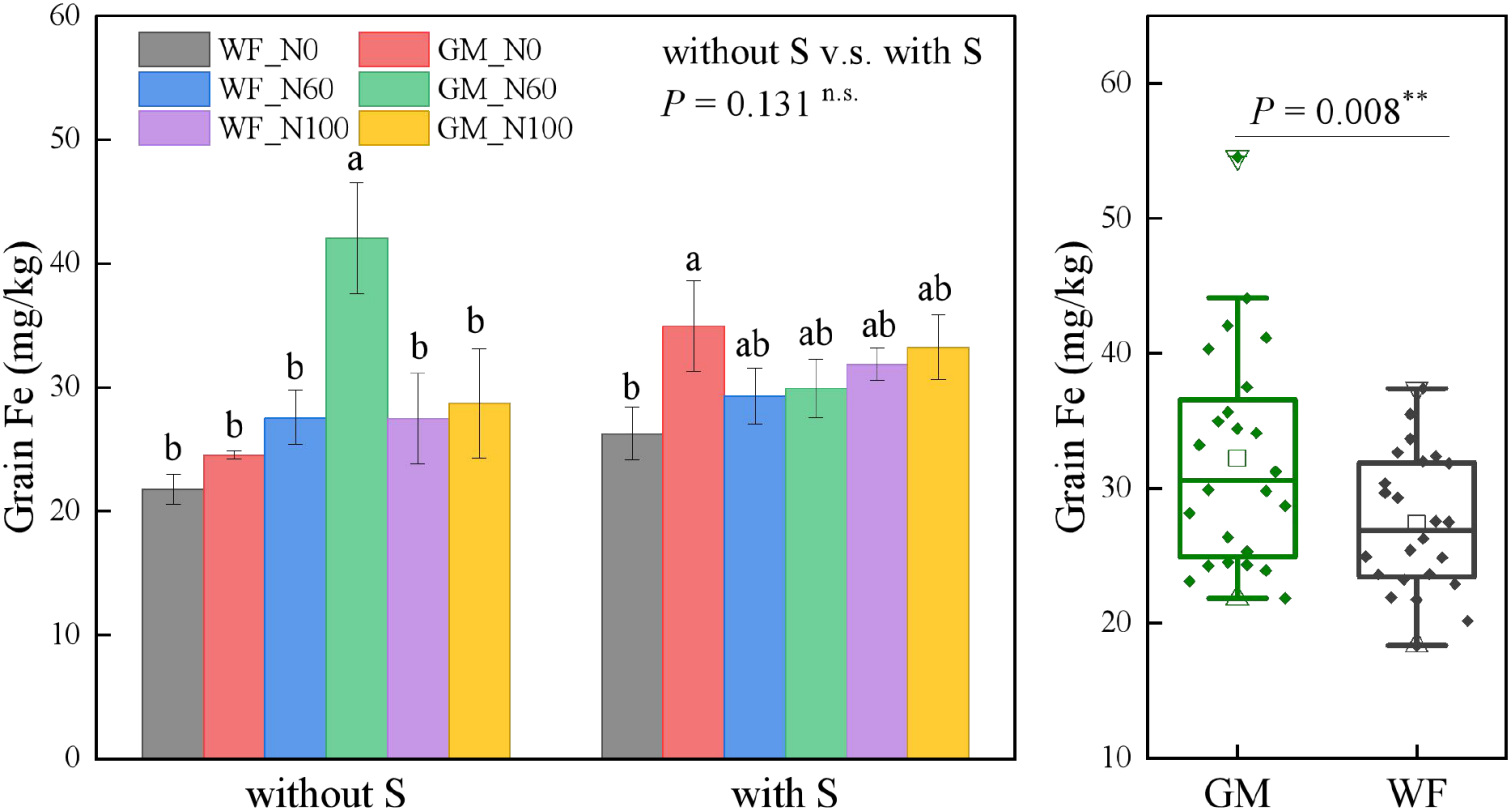
Rice grain iron (Fe) content under the Co-utilization of Milk Vetch and Rice Straw. Note: Without S and with S represent without rice straw return and with rice straw return, respectively. WF_N0, WF_N60, and WF_N100 represent winter fallow with0%, 60%, and 100% conventional N fertilizer, respectively. GM_N0, GM_N60, and GM_N100 represent green manuring with 0%, 60%, and 100% conventional N fertilizer, respectively (*n* = 4). GM represents the combination of all 6 treatments with green manure, and WF represents the combination of all 6 treatments without green manure (*n* = 24). In the box figures, the solid line and dot within each box represent the median and mean values, respectively. The top and bottom edges represent the 75th and 25th percentiles, respectively; the top and bottom error bars represent the 95th and 5th percentiles, respectively; and the top and bottom triangles represent the 99th and 1st percentiles, respectively. * represents *p* < 0.05, ** represents *p* < 0.01. n.s. represent no significant difference (*p* > 0.05).

**Figure 4:**
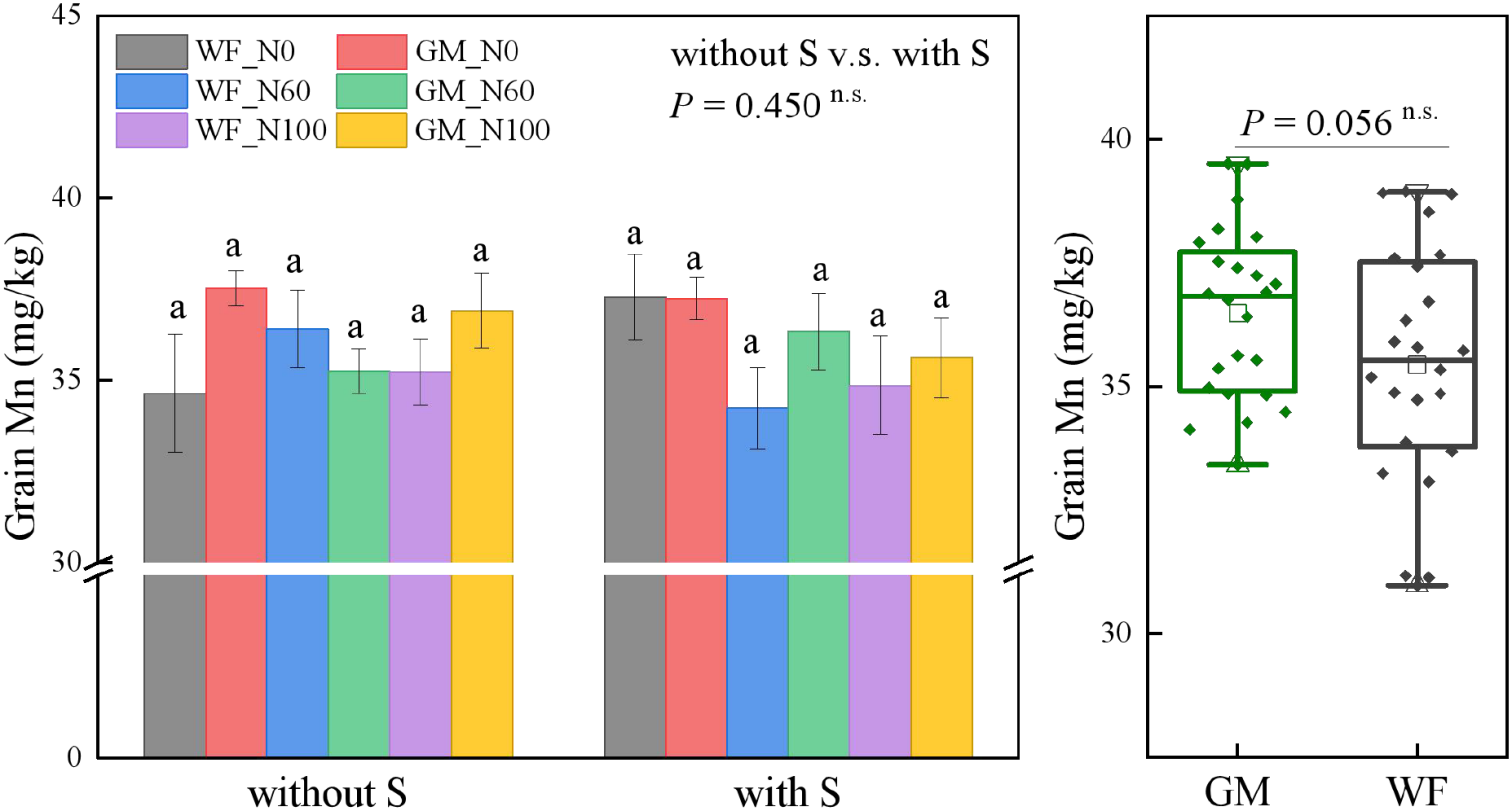
Rice grain manganese (Mn) content under the Co-utilization of Milk Vetch and Rice Straw. Note: Without S and with S represent without rice straw return and with rice straw return, respectively. WF_N0, WF_N60, and WF_N100 represent winter fallow with0%, 60%, and 100% conventional N fertilizer, respectively. GM_N0, GM_N60, and GM_N100 represent green manuring with 0%, 60%, and 100% conventional N fertilizer, respectively (*n* = 4). GM represents the combination of all 6 treatments with green manure, and WF represents the combination of all 6 treatments without green manure (*n* = 24). In the box figures, the solid line and dot within each box represent the median and mean values, respectively. The top and bottom edges represent the 75th and 25th percentiles, respectively; the top and bottom error bars represent the 95th and 5th percentiles, respectively; and the top and bottom triangles represent the 99th and 1st percentiles, respectively. * represents *p* < 0.05, ** represents *p* < 0.01. n.s. represent no significant difference (*p* > 0.05).

The average Fe content in rice grains under GM treatments had significantly increased by 17.75% (*p* < 0.01) relative to that under WF treatments and by 7.80% under straw return treatments relative to that under treatments without straw return. Without straw returning, the Fe content in rice grains under GM_N60 had increased by 93.33%, 71.51%, 52.56%, 52.93%, and 46.48% (*p* < 0.05) relative to those under WF_N0, GM_N0, WF_N60, WF_N100, and GM_N100, respectively. At the same N level, the Fe content in rice grains under GM treatments was higher than that under WF treatments. Under straw return conditions, the differences between treatments were relatively small. At the same N level, GM treatments resulted in higher contents than WF treatments. GMS_N0 resulted in an increase of 33.16% relative to the WFS_N0 treatment, whereas no significant differences were found among other treatments (**Figure 3**). No significant differences in the Mn content in rice grains were found among the treatments: although the average Mn content under GM treatments was slightly higher than that under WF treatments, this difference was not significant (**Figure 4**).

**Figure 5:**
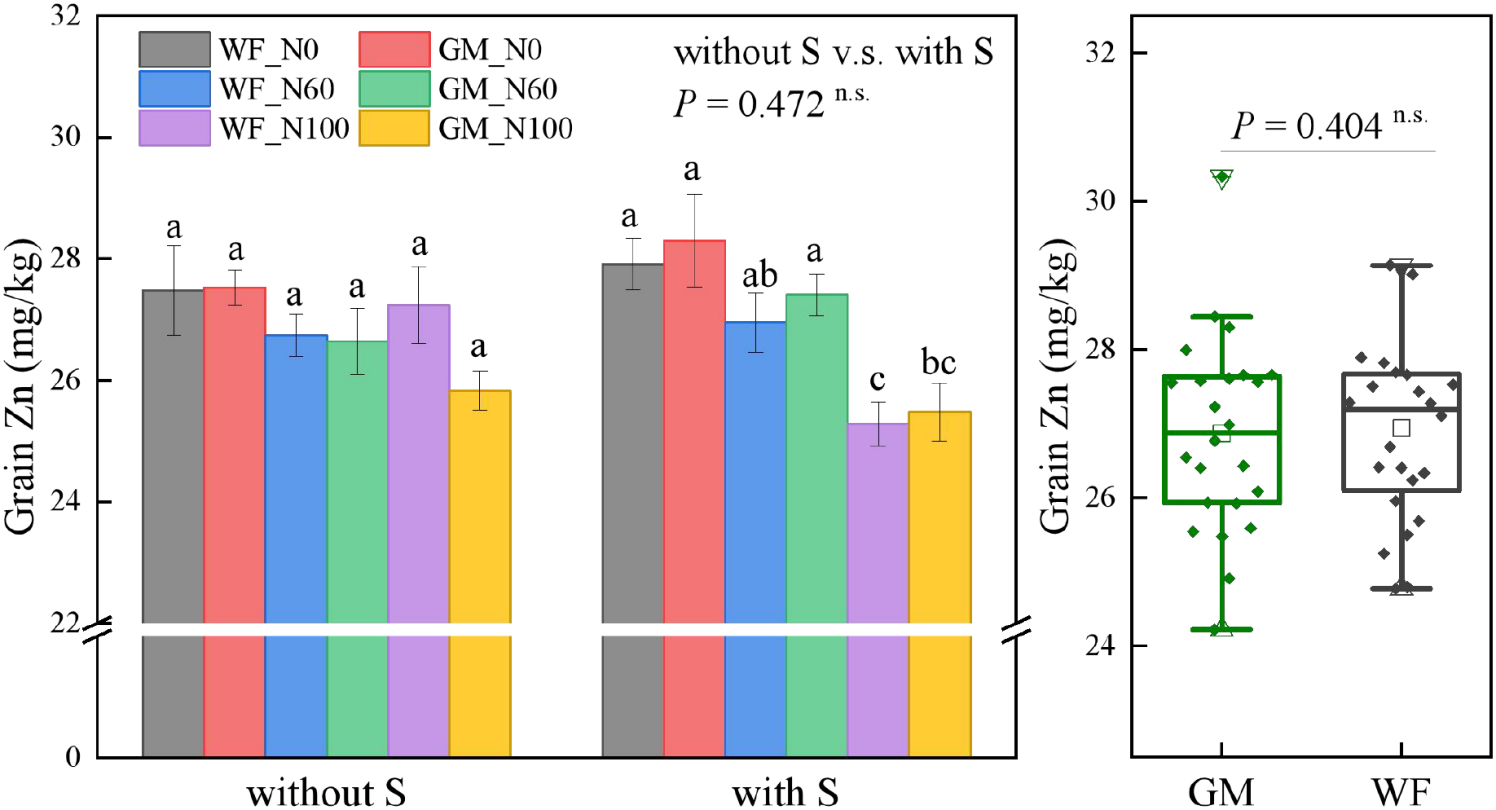
Rice grain zinc (Zn) content under the Co-utilization of Milk Vetch and Rice Straw. Note: Without S and with S represent without rice straw return and with rice straw return, respectively. WF_N0, WF_N60, and WF_N100 represent winter fallow with0%, 60%, and 100% conventional N fertilizer, respectively. GM_N0, GM_N60, and GM_N100 represent green manuring with 0%, 60%, and 100% conventional N fertilizer, respectively (*n* = 4). GM represents the combination of all 6 treatments with green manure, and WF represents the combination of all 6 treatments without green manure (*n* = 24). In the box figures, the solid line and dot within each box represent the median and mean values, respectively. The top and bottom edges represent the 75th and 25th percentiles, respectively; the top and bottom error bars represent the 95th and 5th percentiles, respectively; and the top and bottom triangles represent the 99th and 1st percentiles, respectively. * represents *p* < 0.05, ** represents *p* < 0.01. n.s. represent no significant difference (*p* > 0.05).

The average Zn content in rice grains did not significantly differ between GM and WF treatments nor between treatments with and without straw return. Without rice straw returning, Zn content in rice grains did not significantly differ among treatments. Under straw returning conditions, Zn content under GMS_N0, GMS_N60 increased significantly by 11.91% and 8.38% (*p* < 0.05) compared with that under WFS_N100 (**Figure 5**).

**Figure 6:**
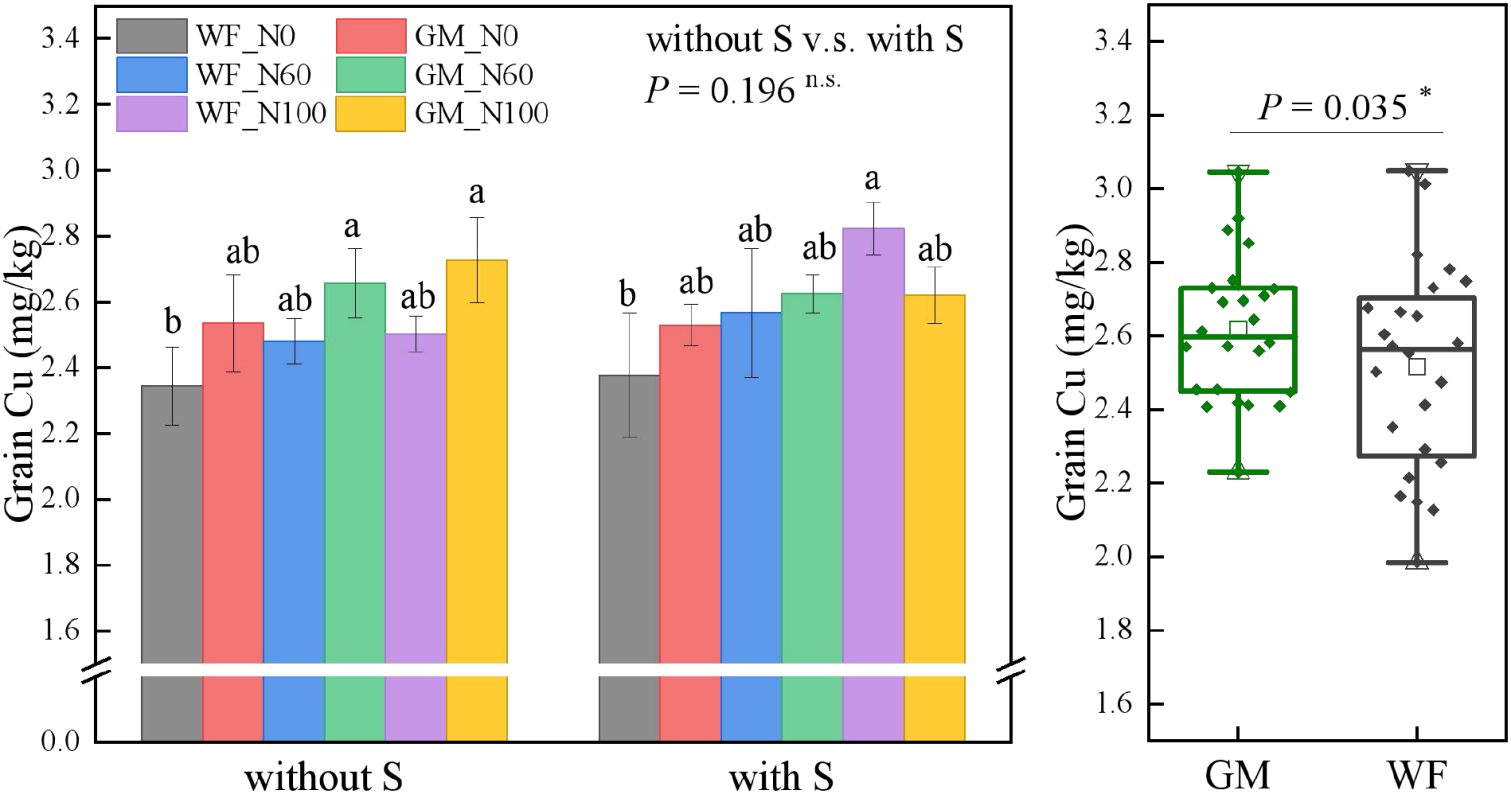
Rice grain copper (Cu) content under the Co-utilization of Milk Vetch and Rice Straw. Note: Without S and with S represent without rice straw return and with rice straw return, respectively. WF_N0, WF_N60, and WF_N100 represent winter fallow with0%, 60%, and 100% conventional N fertilizer, respectively. GM_N0, GM_N60, and GM_N100 represent green manuring with 0%, 60%, and 100% conventional N fertilizer, respectively (*n* = 4). GM represents the combination of all 6 treatments with green manure, and WF represents the combination of all 6 treatments without green manure (*n* = 24). In the box figures, the solid line and dot within each box represent the median and mean values, respectively. The top and bottom edges represent the 75th and 25th percentiles, respectively; the top and bottom error bars represent the 95th and 5th percentiles, respectively; and the top and bottom triangles represent the 99th and 1st percentiles, respectively. * represents *p* < 0.05, ** represents *p* < 0.01. n.s. represent no significant difference (*p* > 0.05).

The average Cu content in rice grains under GM treatments had significantly increased by 3.99% (*p* < 0.05) compared with that under WF treatments. In the absence of straw return and at the same N level, Cu content in rice grains under GM treatments was higher than that under WF treatments, with the Cu content under GM_N60 increasing by 13.29% (*p* < 0.05) compared to WF_N0. Under straw returning conditions, the Cu content under WF_N100 had increased by 18.78% (*p* < 0.05) compared with that under WF_N0 (**Figure 6**).

**Figure 7:**
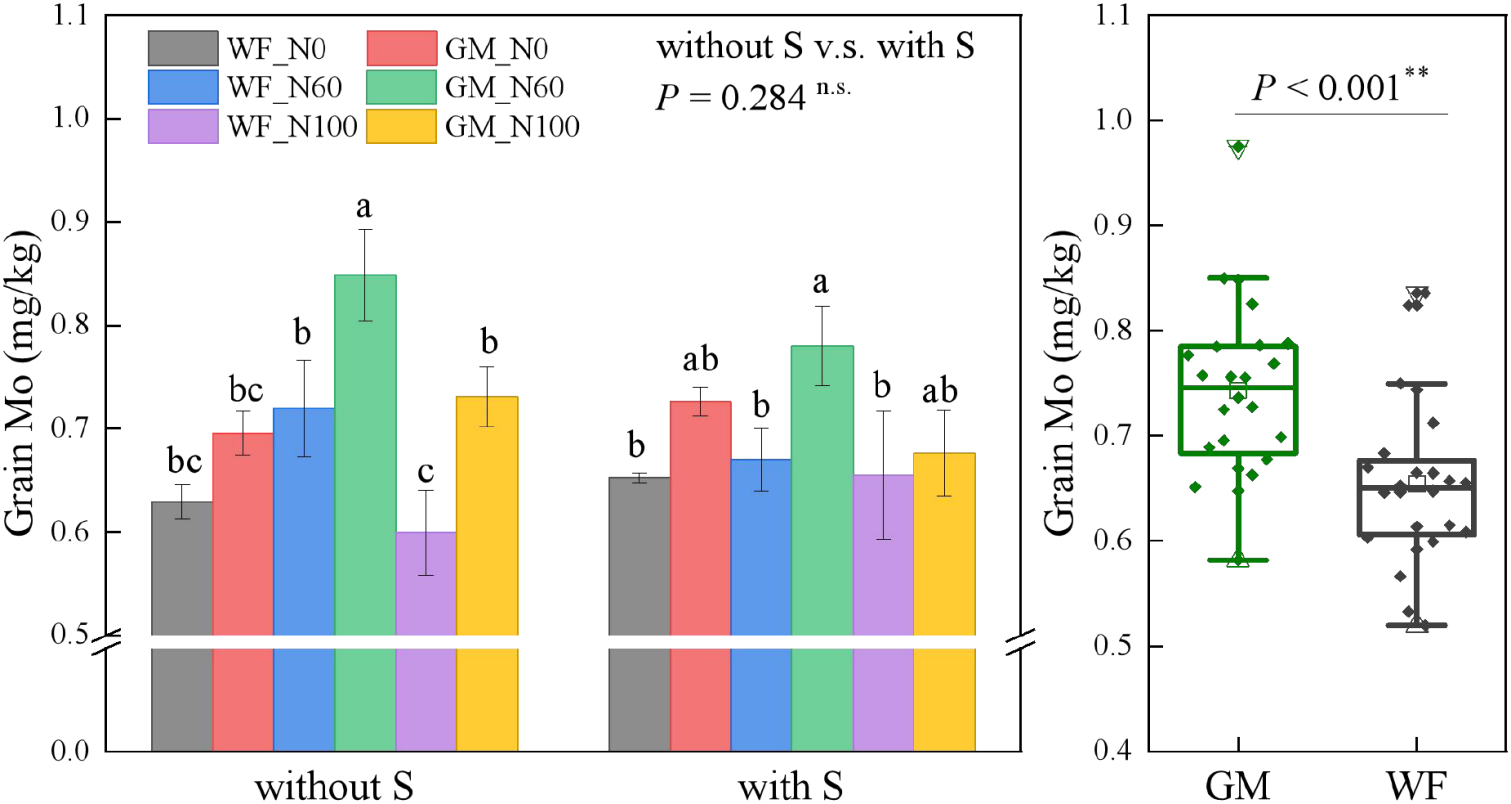
Rice grain molybdenum (Mo) content under the Co-utilization of Milk Vetch and Rice Straw. Note: Without S and with S represent without rice straw return and with rice straw return, respectively. WF_N0, WF_N60, and WF_N100 represent winter fallow with0%, 60%, and 100% conventional N fertilizer, respectively. GM_N0, GM_N60, and GM_N100 represent green manuring with 0%, 60%, and 100% conventional N fertilizer, respectively (*n* = 4). GM represents the combination of all 6 treatments with green manure, and WF represents the combination of all 6 treatments without green manure (*n* = 24). In the box figures, the solid line and dot within each box represent the median and mean values, respectively. The top and bottom edges represent the 75th and 25th percentiles, respectively; the top and bottom error bars represent the 95th and 5th percentiles, respectively; and the top and bottom triangles represent the 99th and 1st percentiles, respectively. * represents *p* < 0.05, ** represents *p* < 0.01. n.s. represent no significant difference (*p* > 0.05).

The average Mo content in rice grains under GM treatments had increased by 13.56% compared with that under WF treatments (*p* < 0.01). In the absence of straw return, at the same N60 and N100 levels, the Mo content in rice grains under GM treatments was higher than that under WF treatments. Mo content was highest under GM_N60 and had increased by 34.83%, 17.94%, and 41.55% (*p* < 0.05) relative to those under WF_N0, WF_N60, and WF_N100, respectively, and by 21.98% and 16.08% (*p* < 0.05) relative to those under GM_N0 and GM_N100, respectively. Under straw returning conditions, Mo content was highest under GMS_N60 and had increased by 19.60%, 16.48%, and 19.12% (*p* < 0.05) relative to those under WFS_N0, WFS_N60, and WFS_N100, respectively (**Figure 7**).

**Figure 8:**
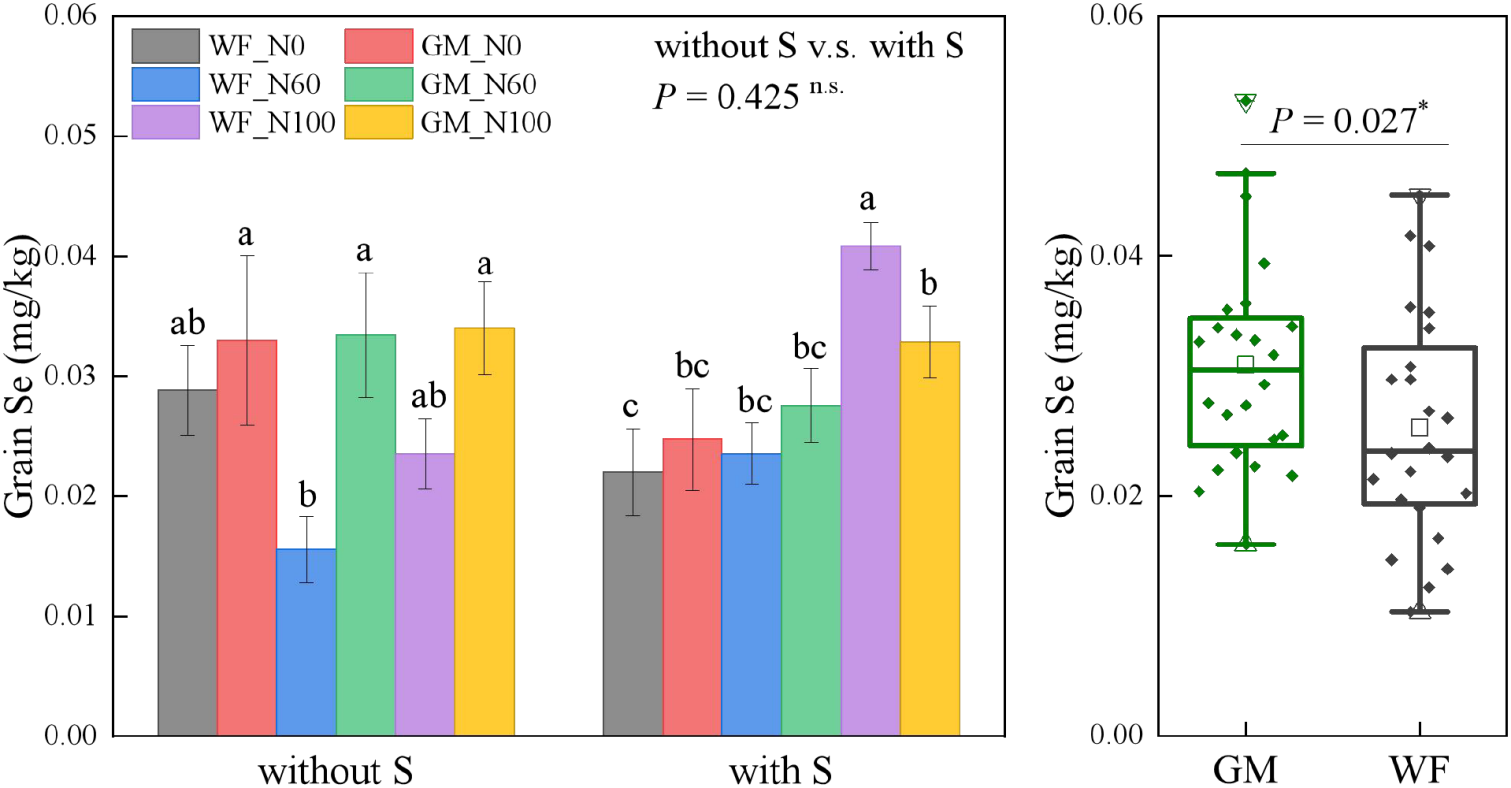
Rice grain selenium (Se) content under the Co-utilization of Milk Vetch and Rice Straw. Note: Without S and with S represent without rice straw return and with rice straw return, respectively. WF_N0, WF_N60, and WF_N100 represent winter fallow with0%, 60%, and 100% conventional N fertilizer, respectively. GM_N0, GM_N60, and GM_N100 represent green manuring with 0%, 60%, and 100% conventional N fertilizer, respectively (*n* = 4). GM represents the combination of all 6 treatments with green manure, and WF represents the combination of all 6 treatments without green manure (*n* = 24). In the box figures, the solid line and dot within each box represent the median and mean values, respectively. The top and bottom edges represent the 75th and 25th percentiles, respectively; the top and bottom error bars represent the 95th and 5th percentiles, respectively; and the top and bottom triangles represent the 99th and 1st percentiles, respectively. * represents *p* < 0.05, ** represents *p* < 0.01.n.s. represent no significant difference (*p* > 0.05).

The average Se content in rice grains under GM treatments had significantly increased by 20.25% (*p* < 0.05) compared to WF treatments (**Figure 8**). Without rice straw returning, the Se contents in rice grains under GM_N0, GM_N60, and GM_N100 were relatively consistent at 0.033, 0.033, and 0.034 mg/kg, respectively. The Se content under GM_N60 had increased by 114.90% (*p* < 0.05) and 42.18% compared with those under WF_N60 and WF_N100, respectively. Under straw return (with S), the Se content under WFS_N100 was the highest at 0.041 mg/kg and that under GMS_N100 had increased by 85.36% (*p* < 0.05) compared with that under WFS_N0.

### 3.4 Silicon content in rice straw under the co-utilization of milk vetch and rice straw.

**Figure 9:**
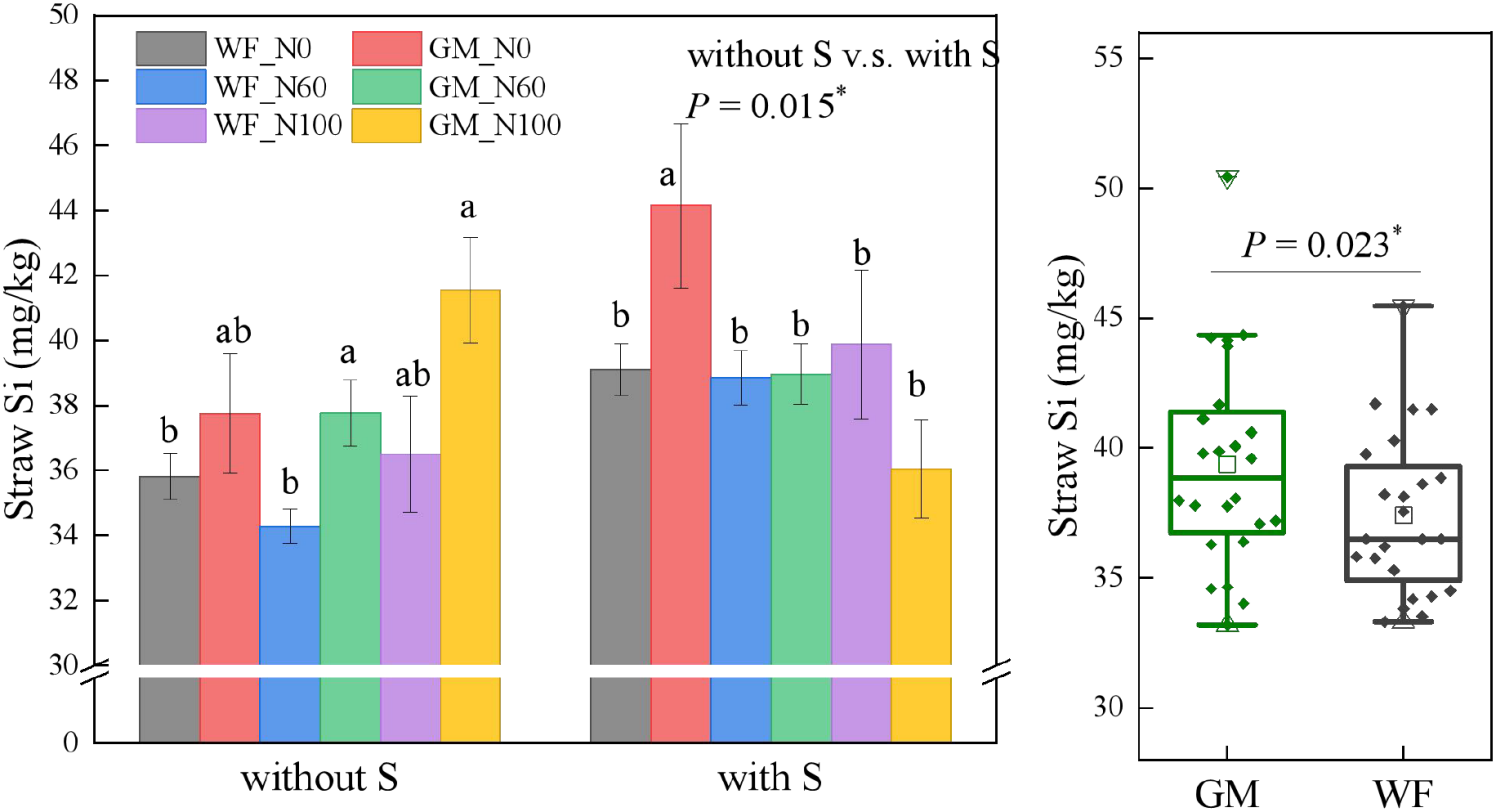
Rice straw silicon (Si) content under the Co-utilization of Milk Vetch and Rice Straw. Note: Without S and with S represent without rice straw return and with rice straw return, respectively. WF_N0, WF_N60, and WF_N100 represent winter fallow with0%, 60%, and 100% conventional N fertilizer, respectively. GM_N0, GM_N60, and GM_N100 represent green manuring with 0%, 60%, and 100% conventional N fertilizer, respectively (*n* = 4). GM represents the combination of all 6 treatments with green manure, and WF represents the combination of all 6 treatments without green manure (*n* = 24). In the box figures, the solid line and dot within each box represent the median and mean values, respectively. The top and bottom edges represent the 75th and 25th percentiles, respectively; the top and bottom error bars represent the 95th and 5th percentiles, respectively; and the top and bottom triangles represent the 99th and 1st percentiles, respectively. * represents *p* < 0.05, ** represents *p* < 0.01. n.s. represent no significant difference (*p* > 0.05).

GM and straw return significantly increased the Si content in rice straw. Si content under GM treatments had significantly increased by 5.24% (*p* < 0.05) compared to WF treatments (**Figure 9**). Moreover, the Si content under straw return treatments had increased by 5.96% (*p* < 0.05) compared with that under treatments without straw return. Without straw returning conditions, the Si content in rice straw under GM_N100 and GM_N60 had increased by 10.12% and 21.16% (*p* < 0.05) respectively compared with that under WF_N60. Under straw returning conditions, Si contents did not significantly differ among different N fertilizer gradients under WF conditions. The Si content in rice straw under the GMS_N0 treatment was significantly higher than those under other treatments and had increased by 12.88%, 13.64%, and 10.72% (*p* < 0.05) compared with those under WFS_N0, WFS_N60, and WFS_N100, respectively, and by 13.30% and 22.46% (*p* < 0.05) compared with those under GMS_N60 and GMS_N100, respectively.

### 3.5 Synergistic or antagonistic relationships among rice elements

**Figure 10:**
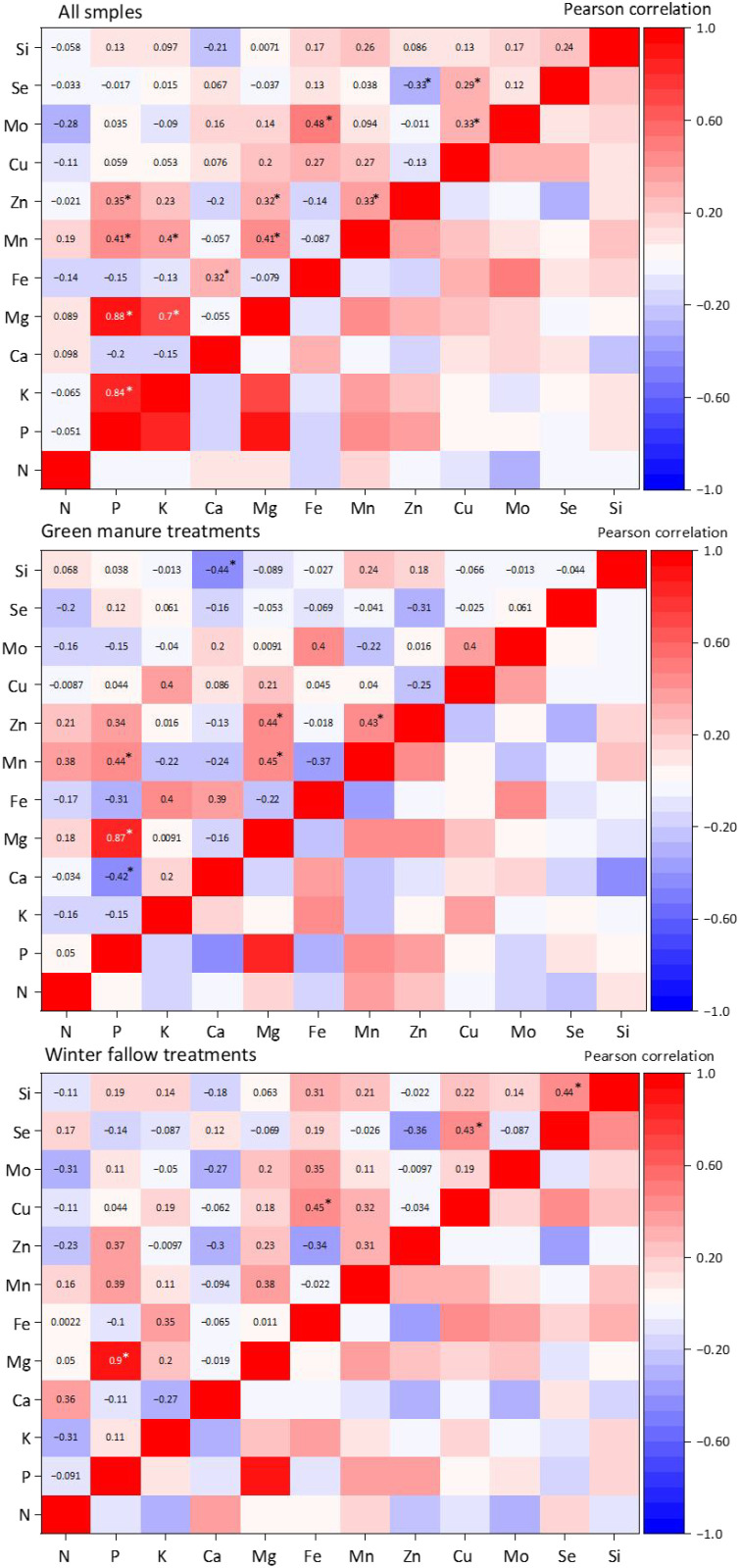
Correlation analysis of various elements in rice under the Co-utilization of Milk Vetch and Rice Straw. GM represents the combination of all 6 treatments with green manure, and WF represents the combination of all 6 treatments without green manure (*n* = 24)* represents *p* < 0.05, ** represents *p* < 0.01.

Pearson correlation analysis was used to clarify the interrelationships among elements (**Figure 10**). The results of the combined analysis of all treatments showed that the P content in rice grains was positively correlated with Mg, Mn, and Zn contents, with correlation coefficients of 0.88, 0.41, and 0.35 (*p* < 0.05), respectively. Mg content was also positively correlated with Mn and Zn contents, with correlation coefficients of 0.41 and 0.32 (*p* < 0.05), respectively. A significant positive correlation was observed between Ca and Fe contents, with a correlation coefficient of 0.32. Fe and Cu contents were positively correlated with Mo content, with correlation coefficients of 0.48 and 0.33 (*p* < 0.05), respectively. Se content was significantly positively correlated with Cu content (0.29) but significantly negatively correlated with Zn content (−0.33).

The separate analysis of GM treatments revealed significant positive correlations between P and Mg, between Mg and Mn and Zn, and between Mn and Zn. These findings were similar to the results of the combined analysis of all treatments. Under GM treatments, negative correlations were observed between P and Ca and between Ca and Si. The separate analysis of WF treatments revealed positive correlations only between P and Mg, between Fe and Cu, between Cu and Se, and between Se and Si. These results indicate that GM treatments promoted synergistic absorption effects among Mg, Mn, and Zn.

## 4 Discussion

### 4.1 Effects of the co-utilization of milk vetch and rice straw on rice yield and nutrient accumulation

Numerous studies and practices have demonstrated that the coutilization of milk vetch and rice straw can increase rice yield and promote nutrient uptake in rice, serving as an important measure for reducing chemical fertilizer application and improving quality and efficiency in paddy fields (Liu et al., 2019a). In this study, the average grain yield under GM treatments had significantly increased by 5.65% compared with that under WF treatments. Furthermore, under rice straw returning conditons, the grain yield under the GM treatments with 40% N reduction was significantly higher than that under the conventional fertilization treatment and WF treatments. Consistent with the findings of previous studies (Liu et al., 2019b; Watanabe et al., 2013; Wang et al., 2022), the above result indicates that under the premise of reducing chemical N fertilizer application by 40%, the synergistic effects of biological N fixation by milk vetch and nutrient release from rice straw can ensure stable and high rice yields.

N uptake in rice grains markedly improved under the co-utilization of milk vetch and rice straw with reduced N application. This improvement may be attributed to the lower C-to-N ratio (C:N) of milk vetch than that of rice straw. The co-utilization of milk vetch and rice straw can effectively regulate the C:N ratio of the returned materials, achieving complementarity between the N released from milk vetch decomposition and that from rice straw mineralization. This phenomenon enables soil N supply to match the N demand of rice throughout the entire growth period well, thereby increasing the final N accumulation (Tang et al., 2014; Cherr et al., 2006). The planting and utilization of GM can activate insoluble P in the soil through root exudates and the regulation of phosphate-solubilizing microbial community functions, increasing the supply of available P in the rhizosphere, thereby elevating P accumulation in rice grains. The combined return of GM and rice straw also promotes K absorption in rice grains. Rice straw itself is rich in K, and its return directly supplements a large amount of K to the soil, meeting the high K demand of rice. In addition, GM optimizes the N–P balance, promoting the distribution of P to grains (Thorup-Kristensen et al., 2003; Zhou et al., 2021).

### 4.2 Co-utilization of milk vetch and rice straw promotes the absorption of secondary and micronutrients in rice grain

Ca act as a structural component of cell walls, maintains cellular structural and functional stability (Montanaro et al., 2015). Mg is closely associated with protein synthesis and rice quality (Li et al., 2022; Hermans et al., 2005). This study found that milk vetch promoted the absorption of Ca and Mg in rice grains likely because substances, such as root exudates produced during GM growth and organic acids released during decomposition, can activate insoluble Ca and Mg in soil colloids through proton exchange and complexation, thereby improving their bioavailability (Schüttelndreier and Falkengren-Grerup, 1999; Kim et al., 2010). Moreover, the growth and development of rice roots are promoted by the improvement in soil physical properties, such as aggregate formation and increased porosity (Liu et al., 2010), resulting from planting and utilizing GM. This effect enhances the absorption efficiency of elements, like Ca and Mg, with low mobility. Studies have indicated that excessive N can inhibit the absorption of Ca by roots (Bonomelli et al., 2021; José, 2021). In this study, the effect of reduced N application in promoting Ca absorption improved likely because the ion competition between ammonium and divalent cations at root absorption sites reduced, further improving Ca absorption efficiency.

Fe participates in chlorophyll synthesis, respiration, and photosynthesis (Becker and Asch, 2005; Cai et al., 2002). Mn facilitates seed development and is a key factor affecting photosynthesis, regulating enzyme activity, and promoting nitrogen (N) metabolism (Yin et al., 2016). Zn is essential for maintaining cell membrane stability and the integrity of plasma membrane function (Cakmak and Kutman, 2018). Cu participates in photosynthesis and respiration (Burkhead et al., 2009).

Winter green manuring promotes the absorption of Fe and Cu in rice grains mainly because the organic acids released during GM decomposition act as chelators to activate insoluble Fe and Mn (Bei et al., 2023; Islam et al., 2019). Additionally, the degradation of organic matter reduces the rhizosphere redox potential, promoting the reductive dissolution of Fe and Mn, thereby increasing their bioavailability. Meanwhile, organic matter complexes with Cu ions to reduce fixation and promote absorption (Bei et al., 2020). Increasing N fertilizer within a certain range can promote the translocation of Fe to grains. However, excessive N fertilizer may reduce the efficiency of Fe accumulation in grains because of dilution effects or interactions between elements. Therefore, moderate N reduction may actually favor the enrichment of Fe in rice. Research shows that in brown rice, an appropriate N application rate (160 kg/ha) can increase the accumulation of Fe, Mn, Cu, and Zn by 28.96%, 41.34%, 58.31%, and 16.0%, respectively, whereas excessive N application may lead to antagonism between elements, a finding that is consistent with the results of this study (Hao et al., 2007).

In rice, Mo deficiency impedes N assimilation and reduces the contents of proteins, particularly those of glutelin and prolamin, thereby affecting nutritional value and cooking characteristics (Kaiser et al., 2005). Se functions to scavenge reactive oxygen species, alleviate oxidative damage during grain filling, reduce chalkiness rate, and improve rice appearance and storage stability (Cartes et al., 2010). Silicon (Si) is a key element influencing the mechanical strength of rice stems; its deficiency predisposes plants to lodging, resulting in yield loss and degraded quality (Rodrigues et al., 2005; Liu et al., 2017a). Mo plays an important role in biological N fixation. It can activate N-fixing microorganisms, such as *Microcoleus* and *Leptolyngbya* of the phylum Cyanobacteria and *Rhizobacter*, *Geobacter*, and *Pseudomonas* of the phylum Proteobacteria, influence crop N metabolism, promote N absorption and transportation, facilitate protein formation, and thereby enhance the N fixation capacity of rhizobia (Ma, 2019; Kaiser et al., 2005). Given the demand for symbiotic N fixation by rhizobia, milk vetch enriches Mo in its tissues, the return of green manure can release organic Mo to supplement the soil-available Mo pool, meanwhile, the improvement of soil organic matter by green manure promotes the transformation of soil Mo from mineral forms to available forms (Gupta and Gupta, 1997). In this study, GM combined with 40% N reduction significantly increased the Mo content in rice grains, verifying the important role of leguminous GM in Mo absorption. Under 40% N reduction, the effect of GM in promoting Mo absorption was optimal mainly because at high N levels, moderate N reduction alleviated the competitive antagonism between nitrate and molybdate ions at absorption channels, favoring Mo absorption. Furthermore, as a key cofactor of nitrate reductase, the moderate N reduction may upregulate its physiological demand for Mo and its transport efficiency to grains to maintain efficient N utilization (Kaiser et al., 2005; Moussa et al., 2021; Liu et al., 2017b). The main mechanism for the increased Se content in rice grains under the GM system is as follows: the plowing and decomposition of GM release large amounts of organic acids and chelating substances, which can activate fixed Se in the soil and promote its transformation into available forms, such as water-soluble and exchangeable states, thereby enhancing the absorption of Se by rice (Hossain et al., 2021; Huang et al., 2022). In this study, the Se content in rice grains under GM treatment increased markedly by 20.25%, indicating that planting and utilizing milk vetch as a GM is a safe and effective technical measure for producing Se-enriched rice.

A noteworthy finding is that under winter green manure without fertilization, the contents of important nutrients such as Se and Mo tended to increase compared with those under winter fallow with conventional fertilization. To some extent, this validates the observation that rice quality is superior in organic production systems without chemical fertilizers, and also provides practical insights for implementing organic rice production under a green manure regime. A possible explanation for this phenomenon is that, unlike the large inputs of chemical nutrients, the high-quality organic materials supplied by green manure provide a more balanced supply of various elements required for rice growth, thereby promoting nutrient uptake and grain quality formation (Gao et al., 2023).

Synergistic or antagonistic relationships exist between different elements. The synergistic absorption effect among Mg, Mn, and Zn enhanced under GM treatments. Different elements have functional coupling effects in the physiological metabolism of rice. Mg serves as a key cofactor for activating ATPase, participating in the regulation of phosphate transmembrane transport. Meanwhile, P is a structural component of the energy carrier ATP (Ahmed et al., 2023). Zn and Mn are important prosthetic groups for various dehydrogenases, carbonic anhydrases, and superoxide dismutases (Sadeghzadeh and Rengel, 2011; Liu et al., 2025), The synergistic supply of these mineral elements and energy carriers (ATP) supports the growth and metabolic demands of rice, therefore, rice growth and energy metabolism are inevitably accompanied with the synergistic absorption and utilization of these elements. The Co-utilization of GM and rice straw enhances soil fertility capacity; regulates microbial activity and metabolic processes; activates fixed nutrients, such as P, K, Fe, Mn, and Zn in the soil; promotes soil nutrient cycling; and improves the nutrient absorption capacity of rice (Wan et al., 2025; Zhang et al., 2025; Shubhiksha et al., 2024).

### 4.3 Co-utilization of milk vetch and rice straw increases the si content in rice straw

The Si content in rice straw directly reflects the status of soil Si supply and the ability of rice to absorb and transport Si. Adequate Si supply improves resistance to biotic and abiotic stresses and promotes balanced nutrient allocation. Moreover, silicon-mediated regulation of carbohydrate metabolism and enzyme activity contributes to optimal starch accumulation and palatability. The results of this study demonstrate that straw return and GM planting significantly increased the Si content in rice straw. As a typical Si-accumulating plant, rice contains large amounts of amorphous Si in its straw. After straw return, phytoliths gradually dissolve under the action of soil microorganisms and hydrolases and release monosilicic acid, which is absorbable by plants (Yang et al., 2025; Marxen et al., 2016). The effect of N fertilizer application on Si content in rice straw is not unidirectional. With straw returning conditions, the Si content in rice straw under GM treatments increased with the rising N gradient likely because supplying an appropriate amount of N fertilizer combined with GM promoted the development of rice roots and the enhancement of root vitality, strengthening the ability of roots to intercept and absorb Si. N is a component of key enzymes in photosynthesis; appropriate N amount can maintain high metabolic activity in plants and promote Si accumulation to a certain extent (Yang et al., 2018; Coquerel et al., 2025; Wang et al., 2017). However, high concentrations of ammonium N can inhibit the activity of root channel proteins in rice, thereby hindering Si absorption. This inhibitory effect may dominate when the Si supply from straw is sufficient and the N level is high, leading to a decrease in Si content. In this study, under straw return, the Si content in rice straw under GM decreased with the increasing N gradient. Conventional N application reduced the Si content in rice straw by 22.46% compared with the no-N treatment. This result indicates that planting and utilizing GM combined with straw return can provide an appropriate amount of N to paddy fields, and reducing N fertilizer application can promote the improvement of rice quality.

Several limitations in this study should be acknowledged. Although variations secondary and micronutrient contents in rice grain and straw were examined under green manuring, but the corresponding analysis with soil element contents was not conducted, leaving the underlying mechanisms of nutrient translocation and accumulation unclear. Future research should therefore combine systematic soil–plant measurements to elucidate the pathways through which green manure regulates nutrient uptake and element partitioning. In particular, theoretical investigations into the synergistic effects of green manure on soil physicochemical properties, microbial activity, and root physiology are needed to establish a robust foundation for optimizing nutrient management in organic rice production systems.

## 5 Conclusion

In the southern Jiangsu rice-growing area of China, the winter planting of milk vetch effectively increased rice yield and nutrient accumulation, the contents of Ca, Fe, Cu, Mn, and Se in rice grain and Si content in rice straw. Simultaneously, it promoted synergistic absorption among Mg, Mn, and Zn, playing an important role in improving rice quality. Co-utilization of milk vetch and rice straw with 40% N reduction had the most significant effect on increasing rice yield and enhancing the content of secondary and micronutrients in rice. This study fills the research gap regarding the effects of planting and utilizing GM on trace elements in rice in the southern Jiangsu rice-growing area, elucidates the role of GM in stabilizing and increasing rice yield and improving rice quality, and provides support for subsequent research on the mechanisms by which GM enhances the nutritional quality of rice.

## Data Availability

The original contributions presented in the study are included in the article/supplementary material. Further inquiries can be directed to the corresponding author.
